# Evaluating adverse drug event reporting in administrative data from emergency departments: a validation study

**DOI:** 10.1186/1472-6963-13-473

**Published:** 2013-11-12

**Authors:** Corinne M Hohl, Lisa Kuramoto, Eugenia Yu, Basia Rogula, Jürgen Stausberg, Boris Sobolev

**Affiliations:** 1 Department of Emergency Medicine, University of British Columbia, 855 West 12th Avenue, Vancouver, BC V5Z 1 M9, Canada; 2 Department of Emergency Medicine, Vancouver General Hospital, 855 West 12th Avenue, Vancouver, BC V5Z 1 M9, Canada; 3 Centre for Clinical Epidemiology & Evaluation, Vancouver Coastal Health Research Institute, 828 West 10th, Vancouver, BC V5Z 1 M9, Canada; 4 Department of Statistics, University of British Columbia, 900 West 10th Ave, Vancouver, BC V5Z 1 M9, Canada; 5 InstitutfürMedizinischeInformationsverarbeitung, Biometrie und Epidemiologie, Ludwig-Maximilians-UniversitätMünchen, München, Germany; 6 School of Population and Public Health, University of British Columbia, Vancouver, BC, Canada

**Keywords:** Adverse drug event, Adverse drug reaction, Administrative data, Emergency department, Validation, Post-market surveillance, Drug safety

## Abstract

**Background:**

Adverse drug events are a frequent cause of emergency department presentations. Administrative data could be used to identify patients presenting with adverse drug events for post-market surveillance, and to conduct research in patient safety and in drug safety and effectiveness. However, such data sources have not been evaluated for their completeness with regard to adverse drug event reporting. Our objective was to determine the proportion of adverse drug events to outpatient medications diagnosed at the point-of-care in emergency departments that were documented in administrative data.

**Methods:**

We linked the records of patients enrolled in a prospective observational cohort study on adverse drug events conducted in two Canadian tertiary care emergency departments to their administrative data. We compared the number of adverse drug events diagnosed and recorded at the point-of-care in the prospective study with the number of adverse drug events recorded in the administrative data.

**Results:**

Among 1574 emergency department visits, 221 were identified as adverse drug event-related in the prospective database. We found 15 adverse drug events documented in administrative records with ICD-10 codes clearly indicating an adverse drug event, indicating a sensitivity of 6.8% (95% CI 4.0–11.2%) of this code set. When the ICD-10 code categories were broadened to include codes indicating a very likely, likely or possible adverse event to a medication, 62 of 221 events were identifiable in administrative data, corresponding to a sensitivity of 28.1% (95% CI 22.3-34.6%).

**Conclusions:**

Adverse drug events to outpatient medications were underreported in emergency department administrative data compared to the number of adverse drug events diagnosed and recorded at the point-of-care.

## Background

Outpatient medication use is common, but may confer health risks that compromise its therapeutic benefits [1-3]. Health risks associated with medications have been shown to vary substantially in clinical practice from those observed in published randomized controlled trials [4,5]. Capturing complete data on adverse drug events observed in clinical practice is important for post-market surveillance, drug regulatory activities, and research in drug safety and effectiveness and patient safety [6-8].

Emergency Departments play a pivotal role in North American healthcare systems [9]. They serve as acute diagnostic and treatment centers for ambulatory patients with unexpected and serious medical problems, as a safety net for the underserved and uninsured, and are an accessible portal of entry into acute care hospitals for sick patients. A growing proportion of urgent outpatient visits occur in emergency departments, and the majority of hospital admissions in the United States occur through emergency departments [9]. Ambulatory patients suffering from serious adverse drug events, the unexpected and unintended complications of medication use, commonly seek care in emergency departments [10-15]. Therefore, emergency department administrative data may offer unique opportunities to track population-level data on clinically significant adverse drug events to outpatient medications for the purposes of surveillance and research [16-20].

Administrative databases are readily available, inexpensive and can provide population-level health data on important health outcomes [8]. However, emergency department administrative data have not been evaluated for their completeness in adverse drug event reporting. Our objective was to determine the proportion of adverse drug events identified at the point-of care in two emergency departments by pharmacists and physicians that were recorded in administrative data.

## Methods

### Study design

We linked data from a prospective observational cohort study to emergency department administrative data [21]. We obtained data on patient demographics, emergency department visits, and adverse drug events to outpatient medications from the prospective study. We used each patient’s unique identifier and emergency department visit date to link the prospective study database with administrative databases to look for records of adverse drug events within the administrative data. The administrative databases used ICD-10 diagnostic codes, and one also recorded chief complaint codes. The University of British Columbia Clinical Research Ethics Board (H10-01632) reviewed and approved the study protocol, and waived the need for informed consent.

### Setting

We used data collected as part of a prospective observational study that was conducted in two tertiary care emergency departments in Canada with a combined census of 145,000 patient-visits per year: Vancouver General Hospital (VGH) and St. Paul's Hospital (SPH).

### Study cohort

Our study cohort consisted of patients enrolled into the prospective study. Patients were eligible for enrolment if they presented to the VGH or SPH emergency departments between July 1, 2008 and January 24, 2009. Research assistants enrolled patients using a previously described systematic patient selection algorithm to generate a representative sample. We included all English-speaking patients who were 19 years of age or older and reported using at least one prescription or over-the-counter medication within two weeks of presentation. In the prospective study, we excluded patients if they were agitated, presented with intentional self-poisoning, had previously been enrolled, presented for a scheduled revisit, had been transferred from another hospital, or left against medical advice or prior to being seen by the physician or pharmacist. When we linked the study databases we subsequently excluded the records of patients with multiple visits on the same day because they resulted in unresolved linkages.

### Identification of adverse drug events at the point-of-care

One of three residency-trained clinical pharmacists who were research assistants in the prospective study and the treating emergency physician assessed each patient for adverse drug events in the emergency department in a manner that was independent and blinded to each other’s assessments using a pre-defined algorithm (Additional file 1): First, the pharmacists evaluated whether or not the patient’s visit was due to an adverse drug event using three adapted causality algorithms [22-24]. The causality algorithms were used to standardize the pharmacists’ assessments, and to ensure that the exacerbation of underlying disease was considered as a cause for any events deemed potentially due to an adverse drug event. Inter-rater reliability of the pharmacists’ assessment using the causality algorithms was 0.75. After the pharmacy assessment was complete, we interviewed the treating emergency physician using a standardized questionnaire to determine the patient’s working diagnosis. When the physician and pharmacist determinations of a patient’s adverse drug event status were concordant (i.e.*,* ADE/ADE or No ADE/No ADE), this was considered the criterion standard. If there was any disagreement between ratings (e.g.*,* ADE/No ADE) or uncertainty (e.g.*,* ADE/Uncertain), an independent committee consisting of a clinical pharmacist and a medical toxicologist (who was also a physician), both of whom were otherwise not involved in the study, adjudicated the cases (See Algorithm for adjudication committee, Additional file 1). If the case definition of adverse drug events was met, we categorized the events according to the Hepler & Strand taxonomy (Additional file 2) [25].

### Identification of adverse drug events in the administrative data

We used administrative data that the Vancouver Coastal Health Authority routinely submits to the Canadian Institute for Health Information (CIHI) National Ambulatory Care Reporting System (NACRS). This database contains data on emergency department visits and includes the patient’s diagnosis at the time of discharge in addition to secondary diagnosis fields. We identified adverse drug events in the administrative data using a list of 650 ICD-10 codes generated through a review of the literature [26] and from Stausberg et al., [27,28] and by searching for relevant chief complaint codes (Additional file 3). Our ICD-10 code set included codes that may indicate a manifestation of an adverse drug event (e.g.*,* K25 Gastric ulcer) or an external cause (e.g.*,* Y40-Y59 Drugs, medicaments and biological substances causing adverse effects in therapeutic use). To search for categories of adverse drug events not classified as adverse drug reactions, we also included Y66 (non-administration of surgical and medical care), T36-50 (poisoning by drugs, including overdose and wrong substance given or taken in error), T88.7-T88.9 (unspecified adverse events of a drug, and unspecified complications of medical care), Y57.9 (complications of medical and surgical care: drug or medicament, unspecified), Y69 (unspecified misadventure during surgical and medical care), and Y88.0 (sequelae of adverse effects caused by drugs in therapeutic use). The likelihood of an ICD-10 code representing an adverse drug event was based on the ICD-10 code description and on clinical reasoning (Table 1).

**Table 1:** Examples of ICD-10 codes indicating adverse drug events within each code category

**Code Category**	**Definition**	**Number of codes in category**	**Examples**	
			**Code**	Code description*
A1	The ICD-10 code description includes the phrase “induced by medication/drug.”	114	J70.2	Acute drug-induced interstitial lung disorders
A2	The ICD-10 code description includes the phrase “induced by medication or other causes.”	81	142.7	Cardiomyopathy due to drugs and other external agents
			T88.7	Unspecified adverse event of drug or medicament
B1	The ICD-10 code dictionary includes the phrase “poisoning by medication.”	134	T36	Poisoning by systemic antibiotics
B2	The ICD-10 code dictionary includes the phrase “poisoning by or harmful use of medication or other causes.”	19	X44	Accidental poisoning by, and exposure to, other and unspecified drugs, medicaments and biological substances
C^§^	Adverse drug event deemed to be very likely although the ICD-10 code description does not refer to a drug.	32	L51.2	Toxic epidermal necrolysis
D^§^	Adverse drug event deemed to be likely although the ICD-10 code description does not refer to a drug.	86	N17	Acute renal failure with tubular necrosis
E^§^	Adverse drug event deemed to be possible although the ICD-10 code dictionary does not refer to a drug.	85	K25	Gastric ulcer
			Y66	Non administration of surgical and medical care

*The code descriptions are from the ICD-10 coding dictionary [29].

^§^The likelihood of the ICD-10 code indicating an adverse drug event was adapted from Stausberg et al., [27,28] and where missing, was determined by two investigators who arrived at their ratings independently, and subsequently obtained consensus through discussion (CH and JS).

### Definitions

Adverse drug events were defined as “untoward and unintended symptoms, signs or abnormal laboratory values arising from the appropriate or inappropriate use of prescription or over-the counter medications” [30-32]. All cases deemed adverse drug events had to be associated with an emergency department visit. Adverse drug events were distinguished from drug-related problems by the presence of untoward and unintended symptoms, signs or abnormal laboratory values. Once identified, adverse drug events were classified into mutually exclusive categories according to their etiology (Additional file 2) [25]. Those due to drug exposure were classified as: (1) adverse drug reactions, defined as noxious and/or unintended responses to medication which occurred despite appropriate drug dosage for prophylaxis, diagnosis or therapy of the indicating medical condition; [32] (2) drug interaction, (3) drug use without indication, or (4) supratherapeutic/high dose. Adverse events related to alcohol abuse and illicit drugs were not considered adverse drug events. Adverse drug events due to lack of exposure to a drug were classified as: (6) subtherapeutic/low dose, (7) need to add drug/untreated indication, (8) wrong drug, or (9) noncompliance/failure to receive drug [11,30,31,33] Cases in which drugs were never considered for use in the first place were not considered adverse drug events. Adverse drug events were categorized into chief-complaint-related events versus those found incidentally. The former were deemed to result in the patient’s presenting complaint (i.e.*,* a non steroidal anti-inflammatory drug leading to a gastrointestinal bleed, in which the patient’s complained of “vomiting blood”). The latter, were not deemed to result in the patient’s presenting complaint (i.e.*,* hydrochlorothiazide leading to an abrupt and significant decline in serum sodium in a patient presenting with a skin infection). The emergency physician assigned an adverse drug event severity category at the point-of-care: A severe event caused death or required admission, a moderate event required a change in medical management, and a mild event required no change in therapy [22-24]. The categorizations of adverse drug events by chief-complaint and severity were independent.

### Analysis

We used descriptive statistics to summarize the baseline characteristics of the patient population. We estimated the proportion of patients with at least one adverse drug event code recorded in the administrative hospital or emergency department database among those who were diagnosed with one or more adverse drug events at the point-of-care. This was estimated as the number of patients with recorded adverse drug event codes in categories A1, A2, B1 or B2 in the administrative database, divided by the number of patients with adverse drug events diagnosed at the point-of-care in the prospective database multiplied by 100. We estimated the proportion of false positives by dividing the number of patients with at least one adverse drug event code recorded in the administrative database and no adverse drug events identified at the point-of-care by the number of all patients without adverse drug events identified at the point-of-care, multiplied by 100. As the likelihood of an ICD-10 code identifying a true adverse drug event varied, we expanded the numerator to include codes in categories C, D, or E.

## Results

### Adverse drug events identified at the point-of-care

**Figure 1:**
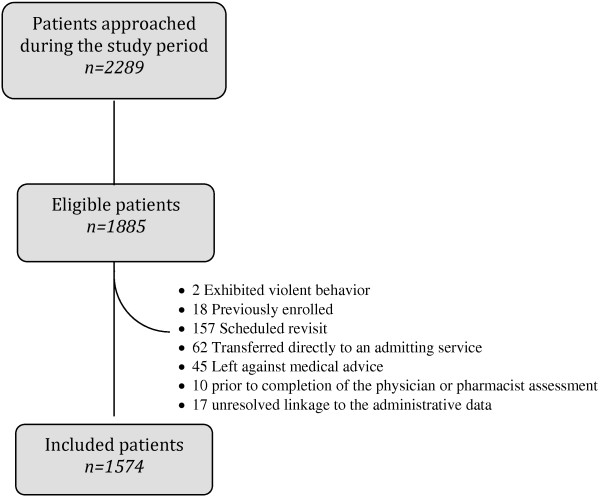
Patient flow.

Among 2289 patients who were approached for enrolment, 1591 met the prospective study’s inclusion and exclusion criteria (Figure 1) [21]. Among these, 17 were excluded because they had multiple visits on the same day in the administrative data leading to unresolved linkages.

The place of presentation was distributed unevenly between the two emergency departments: 1152 (73.2%) patients presented to VGH, and 422 (26.8%) to SPH. The average age was 51.4 years, 51.1% were female, and the median number of prescribed medications was two (Table 2). Among these patients, 221 (14%, 95% confidence interval 12.4–15.8%) were diagnosed with 237 adverse drug events at the point–of–care (Table 3). One hundred and forty six patients (146/221; 66.1% 95% CI 59.6–72.0%) had one adverse drug event related to their chief–complaint, 60 (60/221; 27.1 95% CI 21.7–33.4%) had one incidentally found event, and 15 (15/221; 6.8% 95% CI 4.2–10.9%) had more than one adverse drug event. The most common categories of adverse drug events resulted from adverse drug reactions (74/237; 33.5%; 95% CI 27.6–40.0%) (Table 3). Other categories of adverse drug events were due to noncompliance/failure to receive a drug (62/237; 26.2%; 95% CI 21.0-32.1%), need to add a drug/untreated indication (35/237; 14.8%; 95% CI 10.8-19.9%), subtherapeutic/low dose (27/237; 11.4%; 95% CI 8.0-16.1%), supratherapeutic/high dose (20/237; 8.4%; 5.5-12.7 95% CI), wrong drug (15/237; 6.3%; 95% CI 3.9-10.2%) and drug use without indication (4/237; 1.7%; 95% CI 0.7-4.2%). No adverse drug events were attributed to drug interactions.

**Table 2:** Characteristics of 1,574 patients presenting to the emergency department

**Characteristic**	**All patients (n = 1574)**	**With chief complaint related ADE**	**With incidentally found ADE only**	**Without ADEs**
		**(n = 158)**	**(n = 63)**	**(n = 1,353)**
**Age, yrs, mean (SD)**	51.4 (20.3)	50.8 (20.8)	63.5 (19.7)	50.9 (20.1)
**Female**	805 (51.1%)	76 (48.1%)	26 (41.3%)	703 (52.0%)
**Arrived from, no. (%)**^**†**^				
**Home**	1404 (89.2%)	131 (82.9%)	56 (88.9%)	1217 (89.9%)
**Homeless/Shelter**	64 (4.1%)	14 (8.9%)	2 (3.2%)	48 (3.5%)
**Nursing home**	65 (4.1%)	9 (5.7%)	3 (4.8%)	53 (3.9%)
**Other**	39 (2.5%)	4 (2.5%)	2 (3.2%)	33 (2.4%)
**Canadian triage acuity score, no. (%)**				
**1**	6 (0.4%)	2 (1.3%)	0 (0.0%)	4 (0.3%)
**2**	225 (14.3%)	20 (12.7%)	10 (15.9%)	195 (14.4%)
**3**	693 (44.0%)	77 (48.7%)	36 (57.1%)	580 (42.9%)
**4**	579 (36.8%)	50 (31.6%)	14 (22.2%)	515 (38.1%)
**5**	71 (4.5%)	9 (5.7%)	3 (4.8%)	59 (4.4%)
**Most common chief complaints, no. (%)**				
**Abdominal pain**	157 (10.0%)	4 (2.5%)	9 (14.3%)	144 (10.6%)
**Chest pain**	115 (7.3%)	4 (2.5%)	4 (6.4%)	107 (7.9%)
**Shortness of breath**	96 (6.1%)	14 (8.9%)	5 (7.9%)	77 (5.7%)
**Lower extremity pain**	79 (5.0%)	4 (2.5%)	2 (3.2%)	73 (5.4%)
**Back pain**	64 (4.1%)	4 (2.5%)	0 (0.0%)	60 (4.4%)
**No. comorbid conditions, mean (SD)**	2.1 (2.0)	2.7 (2.2)	3.3 (1.8)	1.9 (2.0)
**Most prevalent comorbid conditions, no. (%)**				
**Hypertension**	382 (24.3%)	43 (27.2%)	36 (57.1%)	303 (22.4%)
**Mood disorder**	201 (12.8%)	35 (22.2%)	7 (11.1%)	159 (11.8%)
**Dyslipidemia**	125 (7.9%)	15 (9.5%)	5 (7.9%)	105 (7.8%)
**Asthma**	120 (7.6%)	20 (12.7%)	6 (9.5%)	94 (6.9%)
**Diabetes Mellitus**	119 (7.6%)	22 (13.9%)	9 (14.3%)	88 (6.5%)
**No. prescribed medications, median (IQR)**	2 (1,5)	4 (2,7)	4 (2,8)	2 (1,5)
**Most commonly prescribed outpatient medications, no. (%)**				
**Acetaminophen with codeine**	195 (12.4%)	29 (18.4%)	8 (12.7%)	158 (11.7%)
**Ramipril**	134 (8.5%)	12 (7.6%)	7 (11.1%)	115 (8.5%)
**Salbutamol**	123 (7.8%)	16 (10.1%)	8 (12.7%)	99 (7.3%)
**Rabeprazole**	117 (7.4%)	12 (7.6%)	10 (15.9%)	95 (7.0%)
**Lorazepam**	106 (6.7%)	7 (4.4%)	6 (9.5%)	93 (6.9%)
**Disposition from the ED, no. (%)**^**‡**^				
**Admitted**	283 (18.0%)	40 (25.3%)	20 (31.7%)	223 (16.5%)
**Home**	1279 (81.3%)	113 (71.5%)	42 (66.7%)	1124 (83.1%)
**Deceased**	3 (0.2%)	0 (0.0%)	0 (0.0%)	3 (0.2%)
**Other**	7 (0.4%)	5 (3.2%)	1 (1.6%)	1 (0.1%)

*Abbreviations*: ADE = adverse drug event, SD = standard deviation, IQR = interquartile range,.

†There were two patients who arrived from an unknown location.

‡There were two patients with unknown disposition from the ED.

**Table 3:** Characteristics of 237 adverse drug events identified at the point-of-care in 1574 emergency department patients

**Characteristic**	**No. (%) of events**	
	**Chief complaint-related**	**Incidentally found**
	(n = 158)	(n = 79)
**Type**		
**Adverse drug reaction**	48 (30.4)	26 (32.9)
**Drug interactions**	0 (0.0)	0 (0.0)
**Drug use without indication**	3 (1.9)	1 (1.3)
**Supratherapeutic/high dose**	12 (7.6)	8 (10.1)
**Subtherapeutic/low dose**	13 (8.2)	14 (17.7)
**Need to add drug/untreated indication**	25 (15.8)	10 (12.7)
**Noncompliance/failure to receive drug**	45 (28.5)	17 (21.5)
**Wrong drug**	12 (7.6)	3 (3.8)
**Severity**		
**Severe**	21 (13.1)	3 (3.8)
**Moderate**	131 (82.9)	71(89.9)
**Mild**	6 (3.8)	5 (6.3)
**Preventability**		
**Preventable**	118 (74.7)	60 (75.9)
**Non-preventable**	40 (25.3)	19 (24.1)

Most adverse drug events (226/237; 95.4%; 95% CI 91.9.0–97.4%) were deemed at least moderate in severity, requiring a change in medical management, consultation, hospital admission, or were life-threatening. The most commonly implicated medications were acetaminophen with codeine (15 events), warfarin (15 events) and phenytoin (9 events).

### Proportion of adverse drug events reported in the administrative databases with codes clearly linking the event to a culprit medication

We found ICD-10 codes that clearly identified an adverse drug event (categories A1, A2, B1 and B2) in 15 of 221 records of patients diagnosed with one or more adverse drug events at the point–of–care (Table 4), corresponding to a sensitivity of 6.8% (95% CI 4.0–11.2%). This code set identified 18 of 1353 records as false positive (1.3%; 95% CI 0.8-2.1%), corresponding to a specificity of 98.7% (95% CI 97.9-99.2%). The positive predictive value of the code set was 45.5% (95% CI 28.1–63.7%), and its negative predictive value 86.6% (95% CI 84.8 – 88.3%).

**Table 4:** Patients with records in the administrative data indicating an adverse drug event among patients with adverse drug events identified at the point-of-care in the emergency department, by category of diagnostic ICD-10 code and type of adverse drug event

ICD-10 code category*	**Any ADE**	**Chief complaint-related**	**Incidentally found**
	(n = 221)	(n = 158)*	(n = 63)**
**Induced or related to a medication (%; 95% CI)**	15 (6.8; 4.0–11.2)	14 (8.9; 5.1–14.7)	1 (1.6; 0.1–9.7)
**Induced or related to a medication,very likely, likely or possible ADE (%; 95% CI)**	62 (28.1; 22.3–34.6)	45 (28.5; 21.7 – 36.3)	17 (27.0; 16.9–39.9)

*Abbreviations*: ICD = international classification of disease; ADE = adverse drug event, CI = confidence interval.

* “Chief complaint-related” refers to patients with at least one chief complaint-related ADE.

** “Incidentally found” refers to patients with incidentally found ADEs only.

Two of 59 adverse drug reactions (3.4%; 95% CI 0.4–11.7%) and 3 of 22 severe adverse drug events (13.6%; 95% CI 2.9–34.9%) were identified as medication-related in the administrative data with code categories A1, A2, B1 or B2, among patients with only one event. Among patients admitted to hospital from the emergency department, 18.2% of adverse drug events were identified with an ICD-10 code clearly linking the event to medication use (Table 5). We found ICD-10 codes that clearly linked a culprit medication to an adverse drug event in 14 of 158 (8.9%, 95% CI 5.1–14.7%) records of patients presenting with a chief complaint-related adverse drug event.

**Table 5:** Patients with records in the administrative data indicating an adverse drug event among patients with adverse drug events identified at the point-of-care in the emergency department, by category of diagnostic code and admission status*

**ICD-10 code category**	**Admitted**	**Discharged**
	(n = 66)	(n = 146)
**Induced or related to a medication (%; 95% CI)**	12 (18.2; 9.8–29.6)	3 (2.1; 0.4–5.9)
**Induced or related to a medication, very likely, likely or possible ADE (%; 95% CI)**	36 (54.6; 41.8–66.9)	26 (17.8; 12.0–25.0)

ADE = adverse drug event; CI = confidence interval.

*Nine patients did not have admission status reported in the administrative data.

### Adverse drug events identified in the administrative data with codes indicating a very likely, likely or possible relationship to a medication

When the ICD-10 code categories were broadened to include codes that very likely, likely or possibly indicated an adverse drug event, we were able to identify 62 codes for adverse drug events in the 221 records of patients diagnosed with one or more adverse drug events at the point–of–care (Table 4). This corresponded to a sensitivity of 28.1% (95% CI 22.3–34.6%) for the broader code set. The positive predictive value of the code set was 27.2% (95% CI 21.5 – 33.5%), and its negative predictive value 88.2% (95% CI 86.3 – 89.9%).

Using the broader code set we identified 23 of 59 adverse drug reactions (39.0%; 95% CI 26.6–52.6%), and seven of 22 severe adverse drug events (31.8%; 95% CI 13.9–54.9%) among patients with one event only. Among admitted patients, 54.6% of adverse drug events were identified with an ICD-10 code indicating a possible, likely, or very likely adverse drug event, or clearly linking the event to medication use (Table 5). The broader code categories identified 45 of 158 (28.5%, 21.7%–36.3%) adverse drug events related to the chief-complaint. This code set incorrectly identified 166 of 1353 records as false positive (12.3%; 95% CI 10.6-14.1%), corresponding to a specificity of 87.7% (95% CI 85.9-89.4%).

The most common culprit medications of all identified adverse drug events (categories A1–E) were olanzapine (five events), and warfarin, phenytoin, vancomycin, glyburide, clopidogrel and aspirin (two events each).

## Discussion

Our objective was to determine the proportion of adverse drug events to outpatient medications resulting in emergency department visits that were reported in administrative data. We found adverse drug events to be underreported in the administrative data of two large Canadian university hospitals, despite using an extensive list of ICD-10 codes. Even when a broad set of ICD-10 codes was used to include diagnoses indicating a very likely, likely or possible relationship with a medication, we were only able to identify 28% of adverse drug events, and 39% of adverse drug reactions. Among patients who required hospital admission, we were able to identify 55% of adverse drug events.

Prescribing medication is the most common medical intervention performed by physicians. Yet, many prescribing decisions are informed by incomplete or conflicting evidence, or by the results of randomized trials that may not be transferrable to clinical practice [7,34,35]. In addition, off-label use of medications and varying compliance behavior of patients contribute to suboptimal treatment outcomes, leading to a growing interest in developing improved methods to capture adverse drug event data from the real-world to generate more robust estimates about the comparative safety and effectiveness of medications, and to develop interventions to improve patient care [6-8,17,18].

In North America, emergency departments offer the majority of healthcare delivered for acute and unexpected medical conditions, including adverse drug events [9]. Therefore, emergency department administrative data may offer unique opportunities to capture data on clinically significant adverse drug events that result from outpatient medication use [9,11-13]. However, before such data are considered for this purpose, they should be evaluated for their completeness.

Our study is the first in the peer-reviewed literature to compare adverse drug event reports in administrative records of emergency department patients with adverse drug events diagnosed at the point-of-care. Prior studies have attempted to validate adverse drug event codes in administrative data by comparing adverse drug event reports in administrative data to events identified by chart review, using electronic trigger methods or between administrative databases [36-41]. Our study differs from these studies in the premise that all clinically significant adverse drug events are recorded in the medical record or identifiable using trigger methods. Indeed, two prior studies support the assumption that 40% of adverse drug events may not be documented in emergency department records [33,42]. Therefore, in order to understand the sensitivity of administrative data and the ICD-10 code set, we derived our criterion standard at the point-of care using a pre-defined algorithm that included assessment by a pharmacist and a physician. We believe that this led to more precise estimates of adverse drug events. We disclosed all adverse drug events suspected in the emergency department to treating physicians (required by Ethics to ensure optimal patient care) prior to coding, thus optimizing the chances of their documentation in the medical chart.

A few studies have examined the sensitivity of adverse drug event codes within the ICD-9 coding system for events that occurred as a result of inpatient medications [38,39]. Hougland et al. found that their code set detected more events than the hospital’s computerized adverse drug event surveillance system, and estimated that 55% of adverse drug events causing hospitalization, and 10% of adverse drug events occurring during the course of hospitalization were identified when compared to medical record review [38]. Leonard et al. found that the sensitivity of their ICD-9 code set varied substantially by the type of adverse drug reaction they searched for, and estimated that the sensitivity of the codes for digoxin and phenytoin related events may be 84% and 86.7% respectively [39]. However, the authors determined the criterion standard retrospectively by chart review in only 19-40% of records, all of which had been were pre-screened using an ICD-9 code set that included the adverse drug reaction codes [39]. This may have falsely elevated the sensitivity of their code set, because the determination of the criterion standard was not independent of the code set they used to identify events.

Only one previous study has evaluated the sensitivity of emergency department data coded in ICD-10 for adverse drug reactions [41]. Wu et al. used CIHI data to compare the emergency department discharge diagnosis with the admitting diagnosis among patients who were admitted to the hospital through emergency departments. The authors’ premise was that in patients admitted to hospital through the emergency department for a diagnosis of an adverse drug reaction, the patient’s emergency department discharge diagnosis and hospital admitting diagnosis should be the same, if adverse drug reactions are appropriately identified, recorded and coded. Using an ICD-10 code set containing 245 codes including the external cause codes Y40-59, Wu et al. found that 15% of emergency department visits for adverse drug reactions leading to hospital admission were coded with the corresponding admitting diagnosis in CIHI. In comparison, in our study including all emergency department patients (not just those admitted to hospital), we were only able to identify 3.4% of adverse drug reactions in the administrative data using our “narrower” code set containing ICD-10 codes categorized as A1, A2, B1 and B2. We believe that this large difference in our estimate of the degree of underreporting may be due to Wu et al.’s comparison of adverse drug reactions coded within one set of administrative data (NACRS) to another (the Discharge Abstract Database), as opposed to our comparison with a prospective standard. This indicates that adverse drug event reporting may be overestimated when reporting is evaluated by comparing between two administrative databases.

The strengths of our study include a rigorous assessment of adverse drug events at the point-of-care before any administrative coding occurred. Both a clinical pharmacist *and* a treating emergency physician assessed all patients in our cohort. The clinical pharmacists in our study evaluated patients independently from physicians, and took their own medical histories, contributing to the accuracy of the available medication information rather than relying on retrospective chart review. Pharmacists documented any suspected adverse drug events in the patients’ records and informed physicians of all potentially missed cases. All cases in which the pharmacists’ and physicians’ assessments of adverse drug events were discordant or uncertain were reviewed and adjudicated by an independent committee consisting of a clinical pharmacist and a medical toxicologist. Another strength of our study includes having conducted a literature review to identify adverse drug event codes in the ICD-10 coding system, reducing the possibility that we underestimated the capacity of the ICD-10 coding system to identify adverse drug events by using too narrow of a code set [26].

The operational definition of adverse drug events remains problematic, as several interpretations of its most common definition “harm caused by the use of a drug” exist [30,31]. We approached our case definition of adverse drug events from the health services research perspective, in which the utilization of the emergency department leading to bed occupancy and incurring cost was the primary end point. Thus, all our cases were associated with an emergency department visit, and we did not capture any “harm” or injury” from illnesses not associated with an emergency department visit. Despite this, not all of the events captured in our study will be of interest from a pharmacovigilance or regulatory body perspective. From the latter perspective, adverse drug reactions, a subset of adverse drug events, are most relevant. Examples of events falling into our case definition that may not be relevant from a pharmacovigilance or regulatory body perspective were the following: the need to add a drug/untreated indication (e.g.*,* lack of anticoagulation therapy leading to stroke in a patient with a previously established diagnosis of atrial fibrillation, a high CHADS2 score and previous documentation of the need for anticoagulation), too high or too low dose (e.g.*,* a reduction in furosemide dose leading to pulmonary edema in a patient with previously controlled congestive heart failure and no alternate explanation), noncompliance/failure to receive a drug (e.g.*,* noncompliance with insulin leading to diabetic ketoacidosis) or wrong drug (e.g.*,* a patient with type II diabetes mellitus with recurrent episodes of hypoglycemia on glyburide). We deemed the inclusion of these types of events important from a health services research perspective, as these types of events have previously been associated with increased health services utilization and cost, [43] and many were classified as preventable [21]. From a patient, clinician and system perspective, the development of methods to identify and monitor these types of events is desirable to generate a factual basis for generating hypotheses about their prevention and to inform health policies to reduce their occurrence. These may include specific actions related to prescribing, administering or monitoring of high-risk medications, or actions targeting specific patient groups. Data on the extent of occurrence and associated burden of events can be used to prioritize actions in a resource-constrained environment to target commonly occurring preventable and costly events. For example, through a recently implemented adverse drug event screening program in the Vancouver Costal Health Authority, through which detailed regional adverse drug event data are collected, our group identified that a large proportion of emergency department visits can be attributed to supratherapeutic/high warfarin dose without any associated bleeding. Identifying the etiologic cause of these visits is informing the development of specific preventative policies within the Health Authority, as well as the discourse between primary care and acute care in terms of the etiology of outpatient adverse drug events and measures for prevention.

In order to ensure that we did not apply too broad of a case definition of adverse drug events, we put mechanisms in place to ensure that events that could be explained by the exacerbation of the patient’s underlying disease or by alternate diagnoses were excluded. These mechanisms included capturing the physician’s working diagnosis, mandating the use of causality algorithms and using an independent adjudication committee. We did not consider the failure to use drug in the first place as an adverse drug event, unless the drug had clearly been documented as being indicated in the patient’s medical record. We put these safeguards in place, as adopting too broad of a case definition and overcalling cases as adverse drug events that might not be, may risk promoting inappropriate use of non-medicinal therapies, and may not serve to promote prudent and rational medication use.

Our study is not without limitations. First, we considered all adverse drug events that had been reported in either primary or secondary diagnostic codes, because some patients presented to the emergency department with more than one adverse drug event, one of which may have been coded under a secondary diagnosis field. Also, some patients may have been diagnosed with more than one diagnosis in the emergency department, one of which was deemed the primary reason for presentation or admission. Therefore, we did not exclude adverse drug event codes that were coded in secondary diagnosis fields in order to avoid underestimating the sensitivity of the administrative dataset and the ICD-10 code set. However, this means that we may have picked up adverse drug events that resulted from in-hospital treatment rather than from outpatient medications. This would have resulted in an overestimation of the sensitivity of the ICD-10 codes. Second, because of the cost and labor involved in establishing a prospective standard for adverse drug events, our sample size is limited. Thus, our study should be regarded as preliminary. Third, our results reflect two Canadian institutions and may not be generalizable to other institutions. Fourth, we expanded our code set to include possible adverse drug events (code categories C, D and E), to allow for better ICD-10 data capture which resulted in a greater proportion of false positives. Fifth, our results may have been influenced by the existing variation in the use of the terminology surrounding adverse drug events [31]. It is possible that physicians were less likely to record (and coders less likely to code) events that they personally felt should not be considered drug-related, even though the presentation met our outcome definition. Finally, we wish to clarify why the number of events listed in this study differs from its parent study [21]: The prospective data used for this study was derived from a prospective observational clinical decision rule derivation study in which we collected data on all outcomes (n = 221). The purpose of the parent study was to derive clinical decision rules to aid health care workers at the point-of-care to identify patients with a broad range of adverse drug events. Yet, this was not possible, likely due to the heterogeneity of the events. Therefore, as stated a priori in the protocol of our parent study, we proceeded to derive clinical decision rules for two narrower categorizations of adverse drug events. Thus, the clinical decision rule derivation study represents a subset of the events analyzed in the present study.

## Conclusion

We found adverse events to outpatient medications resulting in emergency department visits to be underreported in existing administrative data of two large Canadian tertiary care hospitals. The performance characteristics of the code sets examined, in terms of their sensitivity, specificity, positive and negative predictive values, indicate that administrative data alone may not be appropriate as a stand-alone means of identifying adverse drug events in these data. This study may serve as a point of reference for future work in this area, considering the paucity of the literature on evaluating the ICD-10 system and emergency department administrative data for adverse drug event reports. Future research on differential reporting of outpatient adverse drug events in administrative data would be useful to gain a better understanding of those events for which administrative data may have a greater sensitivity [38]. Finally, despite the cost and labor involved in establishing adverse drug event surveillance systems using prospective data, active case finding methods may result in more complete and accurate estimates compared with administrative data.

## Competing interests

None of the authors have identified competing financial or non-financial interests.

## Authors’ contributions

CH, BS and JS have made substantial contributions to the conception and design of the study, CH and EY aquired the data, LK and BR analyzed the data, and all authors interpreted the results of data analysis. CH, LK, BS and JS were involved in drafting and critically reviewing the manuscript. All authors have approved the manuscript in its current form.

## Pre-publication history

The pre-publication history for this paper can be accessed here:

http://www.biomedcentral.com/1472-6963/13/473/prepub

## Supplementary Material

Additional file 1 **ADE Evaluation Algorithm at the Point-of-Care.** In the ED each patient was evaluated by a clinical pharmacist and the treating emergency physician independently, and blinded to each other’s evaluations. The ratings were combined while the patient was still in the ED. If there was any disagreement about the rating (i.e.*,* yes/no, yes/uncertain, no/uncertain, etc.), or if either or both of the evaluations were uncertain, the case proceeded to independent adjudication by a committee consisting of a pharmacist and physician not in any other way involved in the study. Click here for file

Additional file 2 **Adverse drug events were diagnosed only in patients presenting to the emergency department with untoward and unintended symptoms, signs or abnormal laboratory values that arose from appropriate or inappropriate medication use.** Events meeting this case definition were categorized according to the taxonomy of drug-related problems [25]. For all events that were categorized as due to “Need to Add Drug/Untreated Indication” or “Failure to Receive a Drug/Noncompliance” a pre-existing diagnosis had to have been documented prior to the emergency department visit. Failure to use a drug in the first place was not considered an adverse drug event. Asymptomatic drug-related problems were not captured. Adverse drug reactions were defined according to the World Health Organization [31]. Click here for file

Additional file 3 See code set provided in an Excel spreadsheet. Click here for file
