# Supplementary material for: Evaluating adverse drug event reporting in administrative data from emergency departments: a validation study
Source: BMC Health Serv Res. 2013 Nov 12;13:473. doi: 10.1186/1472-6963-13-473 (PMC3842633; doi:10.1186/1472-6963-13-473)
Supplement: Additional file 1 — ADE Evaluation Algorithm at the Point-of-Care. In the ED each patient was evaluated by a clinical pharmacist and the treating emergency physician independently, and blinded to each other’s evaluations. The ratings were combined while the patient was still in the ED. If there was any disagreement about the rating (i.e., yes/no, yes/uncertain, no/uncertain, etc.), or if either or both of the evaluations were uncertain, the case proceeded to independent adjudication by a committee consisting of a pharmacist and physician not in any other way involved in the study. [file 1472-6963-13-473-S1.docx]

**Additional file 1**

**ADE Evaluation Algorithm at the Point-of-Care.**

In the ED each patient was evaluated by a clinical pharmacist and the treating emergency physician independently, and blinded to each other’s evaluations. The ratings were combined while the patient was still in the ED. If there was any disagreement about the rating (*i.e.,* yes/no, yes/uncertain, no/uncertain, etc.), or if either or both of the evaluations were uncertain, the case proceeded to independent adjudication by a committee consisting of a pharmacist and physician not in any other way involved in the study.

After ED visit:

All other ratings

Yes/Yes ADE

No/No ADE

**Evaluation by a Pharmacist:**

Using 3 causality algorithms, combined with their global ADE assessment:

Yes/No/Uncertain

**Standard Evaluation by the Treating Physician**: Physicians were interviewed at the end of the ED visit to see if they diagnosed an ADE:

Yes/No/Uncertain

**Final ADE Rating by Consensus**

Feedback to treating physicians about all ADEs that may have been missed

In the ED:

Algorithm for adjudication committee:

1. Each rater independently evaluated the case and rated the likelihood that the complaint/problem was related to medication use:

1. No evidence that symptom was due to treatment.

2. Little evidence that symptom was due to treatment.

3. Symptom was possibly due to treatment but more likely due to disease.

4. Symptom was possibly due to treatment and more likely due to treatment than disease.

5. Symptom was probably due to treatment.

6. Symptom was definitely due to treatment.

1. Each rater’s score was recorded. If all ratings were >4, the case was considered an ADE. If all ratings were one to three the case was not considered an ADE.
2. If all ratings were the same, that number was the final rating for that patient and problem.
3. If the ratings were different, the case was discussed until consensus was reached on the final rating. The final consensus rating determined whether or not the case was considered an ADE (score 4-6) or not (score 1-3).

**Final Rating by Adjudication**
