# Supplementary material for: Evaluating adverse drug event reporting in administrative data from emergency departments: a validation study
Source: BMC Health Serv Res. 2013 Nov 12;13:473. doi: 10.1186/1472-6963-13-473 (PMC3842633; doi:10.1186/1472-6963-13-473)
Supplement: Additional file 2 — Adverse drug events were diagnosed only in patients presenting to the emergency department with untoward and unintended symptoms, signs or abnormal laboratory values that arose from appropriate or inappropriate medication use. Events meeting this case definition were categorized according to the taxonomy of drug-related problems [25]. For all events that were categorized as due to “Need to Add Drug/Untreated Indication” or “Failure to Receive a Drug/Noncompliance” a pre-existing diagnosis had to have been documented prior to the emergency department visit. Failure to use a drug in the first place was not considered an adverse drug event. Asymptomatic drug-related problems were not captured. Adverse drug reactions were defined according to the World Health Organization [31]. [file 1472-6963-13-473-S2.docx]

**Additional file 2**

Adverse drug events were diagnosed only in patients presenting to the emergency department with untoward and unintended symptoms, signs or abnormal laboratory values that arose from appropriate or inappropriate medication use. Events meeting this case definition were categorized according to the taxonomy of drug-related problems.[25] For all events that were categorized as due to “Need to Add Drug/Untreated Indication” or “Failure to Receive a Drug/Noncompliance” a pre-existing diagnosis had to have been documented prior to the emergency department visit. Failure to use a drug in the first place was not considered an adverse drug event. Asymptomatic drug-related problems were not captured. Adverse drug reactions were defined according to the World Health Organization.[44]

| **Definition** | **Description** |
| --- | --- |
| Drug Interaction | Bioavailability of drug is altered |
|  | Effect of drug has been altered due to enzyme inhibition/induction from another drug |
|  | Effect of drug altered due to substance in food |
|  | Effect of drug has been altered due to displacement from binding sites by another drug |
|  | Laboratory test result has been altered due to presence of drug |
| Drug Use Without Indication | No medical indication for a drug |
|  | Receiving drug chronically which was intended for acute condition |
|  | Addiction/dependence to a medication |
|  | Condition can be more appropriately treated by non-drug therapy |
|  | Receiving a drug that is causing an adverse event to treat an avoidable adverse drug reaction |
|  | Duplicate therapy |
|  | Taking multiple drugs for a condition for which monotherapy is effective |
| Supratherapeutic/High Dose | Dose is too high |
|  | Patient’s serum drug concentration is above desired therapeutic range |
|  | Drug, dose, route or formulation conversions were inadequate |
|  | Drug dose was increased too rapidly |
|  | Accidentally or intentionally ingesting a toxic amount of drug |
| Wrong Drug | Risk for toxicity exists with current drug therapy (contraindication, precautions) |
|  | Cannot take the drug product (*i.e.,* swallow, taste, administer) |
|  | Dosage form is inappropriate |
|  | Drug therapy is known to be ineffective for this indication |
|  | Current drug therapy is not the most effective for the indication being treated |
|  | Patient has become refractory to present drug therapy |
|  | Drug therapy is effective for this indication, but not effective in this patient and needs to be substituted |
|  | Has an infection involving organisms that are resistant to drug |
| Subtherapeutic/Low Dose | Dose is too low to produce desired response for this patient |
|  | Patient’s serum drug concentration is below desired therapeutic range |
|  | Drug, dose, route or formulation conversions were inadequate |
|  | Drug therapy was discontinued before condition treated |
|  | Drug dose is decreased too rapidly |
| Need to Add Drug/  Untreated Indication | Has previously diagnosed disease which requires drug therapy, but is not receiving it |
|  | Taking drug for valid indication, but causes side effects requiring prophylactic therapy that the patient is not receiving |
|  | Requires synergistic drug therapy to potentiate effect of current drug therapy |
| Noncompliance/Failure to Receive Drug | Not taking drug to a previously established condition due to lack of understanding or economic constraints |
|  | Medication error (prescribing, dispensing, administration, monitoring) was made |
|  | Patient is not compliant (not taking according to instructions) |
|  | Required drug that is clearly indicated is not available and has not been substituted with an appropriate alternative |
